# A double blind randomized controlled trial investigating efficacy and safety of varenicline for smoking cessation in patients with type 2 diabetes: study protocol

**DOI:** 10.1007/s11739-021-02684-1

**Published:** 2021-03-18

**Authors:** C. Russo, P. Caponnetto, F. Cibella, M. Maglia, A. Alamo, D. Campagna, L. Frittitta, M. Di Mauro, C. Leotta, E. Mondati, A. Krysiński, E. Franek, R. Polosa

**Affiliations:** 1 Ambulatorio Di Diabetologia, UOC Medicina Interna E D’Urgenza, Policlinico Universitario, AOU “Policlinico-V. Emanuele”, Catania, Italy; 2 Centro Per La Prevenzione E Cura del Tabagismo (CPCT), Azienda Ospedaliero-Universitaria “Policlinico-V.Emanuele” Dell’Università Di Catania, Via S. Sofia 78, 95123 Catania, Italy; 3 Institute for Biomedical Research and Innovation, National Research Council of Italy, Palermo, Italy; 4 Centro Diabetologico - UOC Andrologia Ed Endocrinologia, Policlinico Universitario, AOU “Policlinico-V. Emanuele”, Catania, Italy; 5 UOC MCAU, University Teaching Hospital “Policlinico-Vittorio Emanuele”, University of Catania, Catania, Italy; 6 Centro Per Il Diabete E L’Obesità - UOC Endocrinologia, Ospedale Garibaldi Nesima, ARNAS Garibaldi, Catania, Italy; 7 Center of Excellence for the Acceleration of HArm Reduction (CoEHAR), Dipartimento Di Medicina Clinica E Sperimentale, Università Di Catania, Catania, Italy; 8 Ambulatorio Di Diabetologia, Ospedale V. Emanuele, AOU “Policlinico-V. Emanuele”, Catania, Italy; 9 Ambulatorio Di Diabetologia - UOC Geriatria, Ospedale Cannizzaro, Catania, Italy; 10 UOS Sorveglianza Delle Complicanze Delle Malattie Metaboliche, Ospedale S. Marta, AOU “Policlinico-V. Emanuele”, Catania, Italy; 11 Mossakowski Clinical Research Centre, Polish Academy of Sciences, Warsaw, Poland; 12 Department of Internal Diseases, Endocrinology and Diabetology, Central Clinical Hospital MSWiA, Warsaw, Poland

**Keywords:** Randomized controlled trial, Smoking cessation, Varenicline, Diabetes mellitus

## Abstract

Reducing exposure to cigarette smoke is an imperative for public health and for diabetic patients. Patients with diabetes who continue to smoke face challenges at quitting and the delivery of effective smoking cessation interventions is a major unmet need. The high-affinity α4β2 nicotinic acetylcholine receptor partial agonist varenicline in combination with counseling is effective for smoking cessation, but evidence in patients with diabetes is limited. A clinical trial of varenicline targeted specifically at smokers with T2DM is warranted. This randomized, double blind, placebo-controlled trial will be the first study to test efficacy and safety of varenicline in smokers with type 2 diabetes mellitus (T2DM) over the course of 52 weeks. We hypothesize that varenicline treatment (1 mg BID, administered for 12 weeks) would increase quit rates, maintain smoking abstinence up to 1 year after treatment, and be well-tolerated in T2DM smokers intending to quit. Efficacy end points will include carbon monoxide–confirmed continuous abstinence rate (CAR) and 7-day point prevalence of abstinence. The results of this RCT will help inform medical/health authorities and physicians worldwide whether an optimally varenicline-treated cohort of T2DM patients who smoke will experience significant success rates, without significant side effects.

Trial registration NCT01387425 (https://clinicaltrials.gov/ct2/show/NCT01387425).

## Introduction

Diabetes mellitus (DM) is a condition in which high blood sugar levels persist over a prolonged period of time. The noxious effect of chronic exposure of high sugar levels on the endothelium can cause macrovascular (coronary artery disease, stroke and peripheral arterial disease) or microvascular complications (retinopathy, nephropathy, and diabetic neuropathy) in individuals with type 2 diabetes mellitus (T2DM) [1].

Given that exposure to cigarette smoke is also associated with vascular damage, endothelial cell dysfunction and clotting activation [2, 3], it is not surprising that the combined injurious effects of high blood glucose together with cigarette smoke is likely to cause the accelerated course of vascular complications as well as sexual dysfunction in diabetic patients who smoke [2–4]. Indeed, cigarette smoking has been shown to increase the risk of microvascular and macrovascular complications as well as mortality in patients with T2DM [5–8]. Consistent with these observations, quitting smoking decreases this excess risk substantially [8–10] and improves sexual health [11]. If reducing exposure to cigarette smoke is an imperative for public health, it is even more so for patients with T2DM [12].Currently approved smoking cessation medications in combination with counseling have been shown to double or triple quit rates [13, 14], but the evidence for effective cessation interventions in patients with diabetes is limited [15]. Of note, smoking prevalence in individuals with diabetes continues to be comparable to that found in the general population [16] with declines that are significantly lesser among individuals with diabetes than without diabetes [17]. Consequently, one of the major unmet needs for patients with diabetes who continue to smoke is the delivery of effective smoking cessation interventions.

The high-affinity α4β2 nicotinic acetylcholine receptor partial agonist varenicline is hypothesized to reduce craving and withdrawal symptoms by stimulating dopamine release through its agonist property and to decrease the reinforcing effects of smoking by blocking nicotine binding through its antagonist property [18]. Randomized controlled trials of varenicline (1 mg BID) administered for 12 weeks along with brief smoking cessation counseling have been shown to significantly increase quit rates in smokers with no clinically significant medical conditions [19, 20], as well as smokers with cardiovascular disease [21], chronic obstructive pulmonary disease (COPD) [22], or depression [23]. In light of its efficacy, a clinical trial of varenicline targeted specifically at smokers with T2DM is deemed warranted.

Therefore, we have designed the first randomized, double blind, placebo-controlled study of efficacy and safety of varenicline (1 mg BID, administered for 12 weeks and followed to week 52) in smokers with T2DM. We hypothesized that varenicline treatment would increase quit rates versus placebo, maintain smoking abstinence up to 1 year after treatment, and be well-tolerated among diabetic smokers.

## Methods

### Study participants

Adult smokers receiving regular treatment at diabetic outpatient clinics for T2DM will be screened for the study. To be eligible they will have to satisfy the following criteria:

#### Inclusion criteria

- Type 2 diabetic patients (≤ 75 years of age, but > 18 years of age) who met the ADA diagnostic criteria [24]
- T2DM had been diagnosed for > 12 months
- With 7.0% ≤ HbA1c ≤ 12.0%
- Regularly smoking ≥ 10 cigs/day during the past year (with no period of abstinence greater than three months in the past year)
- Willing to quit. This will be verified by the answer ‘‘YES’’ to both questions ‘‘Do you intend to quit in the next 30 days?’’ and ‘‘Are you interested in taking part in a smoking cessation program?’’
- Females of nonchildbearing potential (surgically sterilized or at least 2 years postmenopausal) who are not nursing may be included. Females of childbearing potential may be included provided that they are not pregnant, not nursing, and are practicing effective contraception.
- Subjects must be able to be outpatients and be assessed in a clinic setting.
- Participating subjects must be able to provide written informed consent.

#### Exclusion criteria

- Subjects currently or within the past 12 months requiring treatment for depression. Subjects with a past or present history of panic disorder, psychosis, or bipolar disorder.
- Subjects with a current or recent (within the past 12 months) history of alcoholism.
- Subjects with a requirement to use other medications during the study that might interfere with the evaluation of the study drug (e.g., nicotine replacement therapy).
- Subjects with a body mass index (BMI) less than 18.5 or greater than 35.
- Subjects with evidence or history of clinically significant allergic (except for seasonal allergies at time of dosing), endocrine, gastrointestinal, hematological, hepatic, neurologic, pulmonary, or renal disease or a history of cancer (excluding treated basal cell carcinoma and squamous cell carcinoma). Exceptions to this exclusion may include subjects with a history of mild COPD, and stable thyroid disease.
- Subjects with a history of clinically significant cardiovascular disease. In addition, subjects with uncontrolled hypertension or a screening or baseline systolic blood pressure greater than 160 mm Hg or a diastolic blood pressure greater than 95 mm Hg will be excluded.

The study adheres to the ethical principles of the Declaration of Helsinki and to the International Conference on Harmonization Good Clinical Practice guidelines. The study has been reviewed and approved by the Ethical Review Board of the leading site at the Azienda Ospedaliero Universitaria “Policlinico-V. Emanuele”, Università di Catania, Italy (approval reference number: 009711) and participants will give written informed consent prior to participation in the study. The study has been registered in ClinicalTrial.gov with Trial registration ID: NCT01387425 (https://clinicaltrials.gov/ct2/show/NCT01387425).

### Study design

**Fig. 1:**
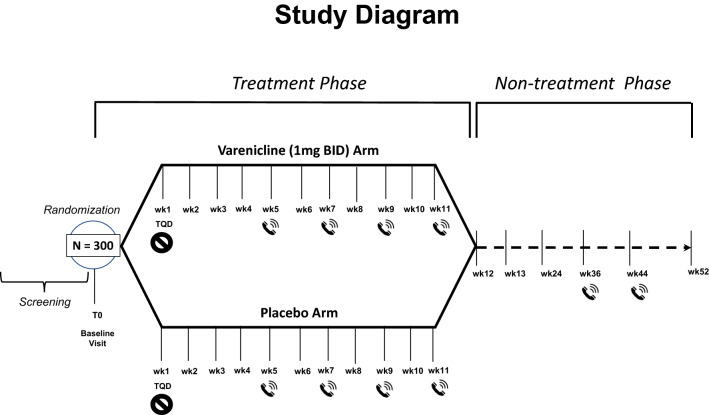
Study design of a double blind placebo controlled randomized clinical trial of varenicline in diabetic smokers

**Fig. 2:**
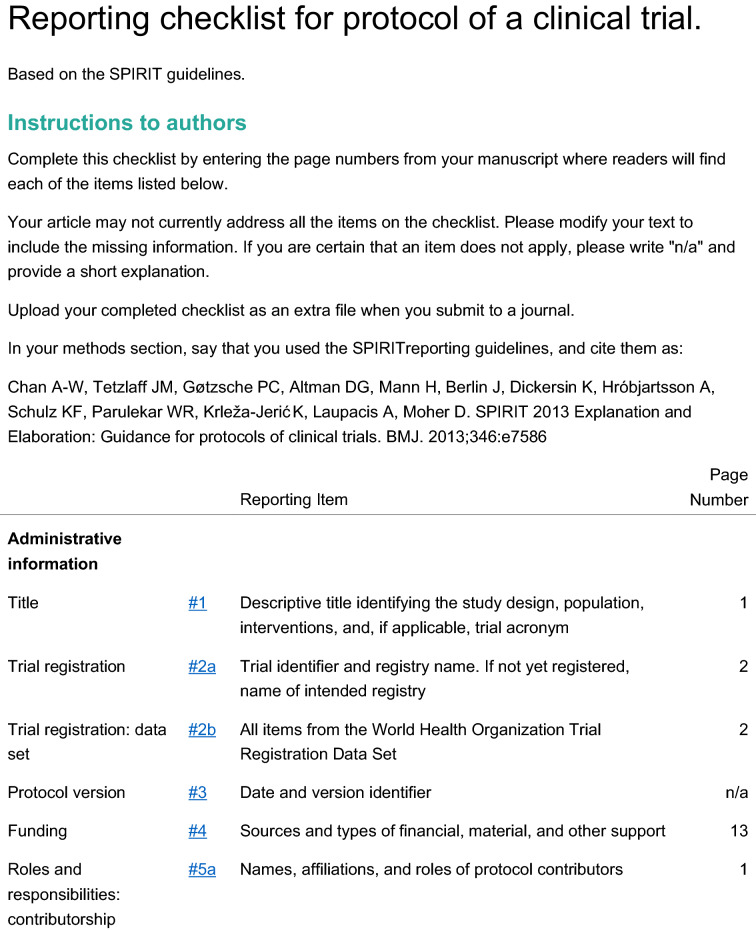

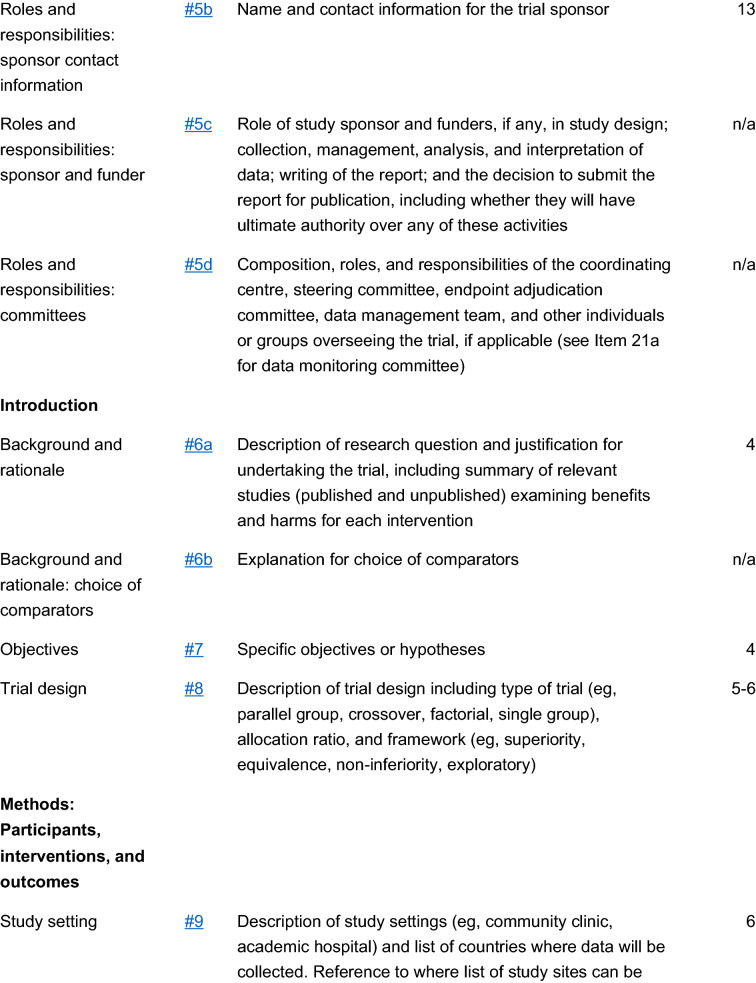

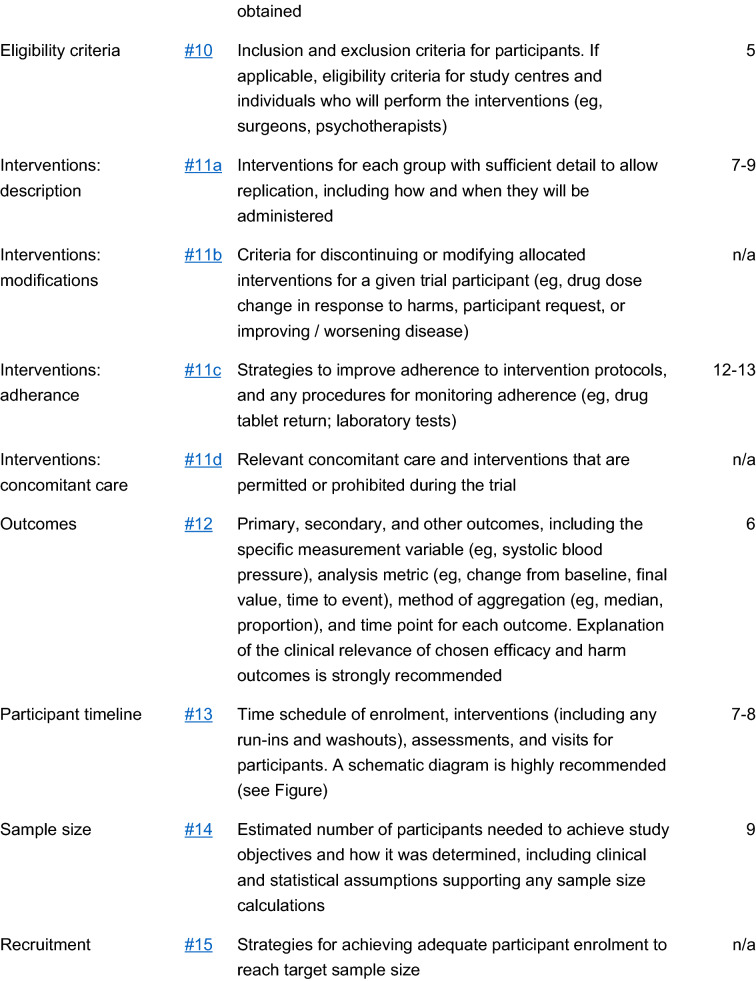

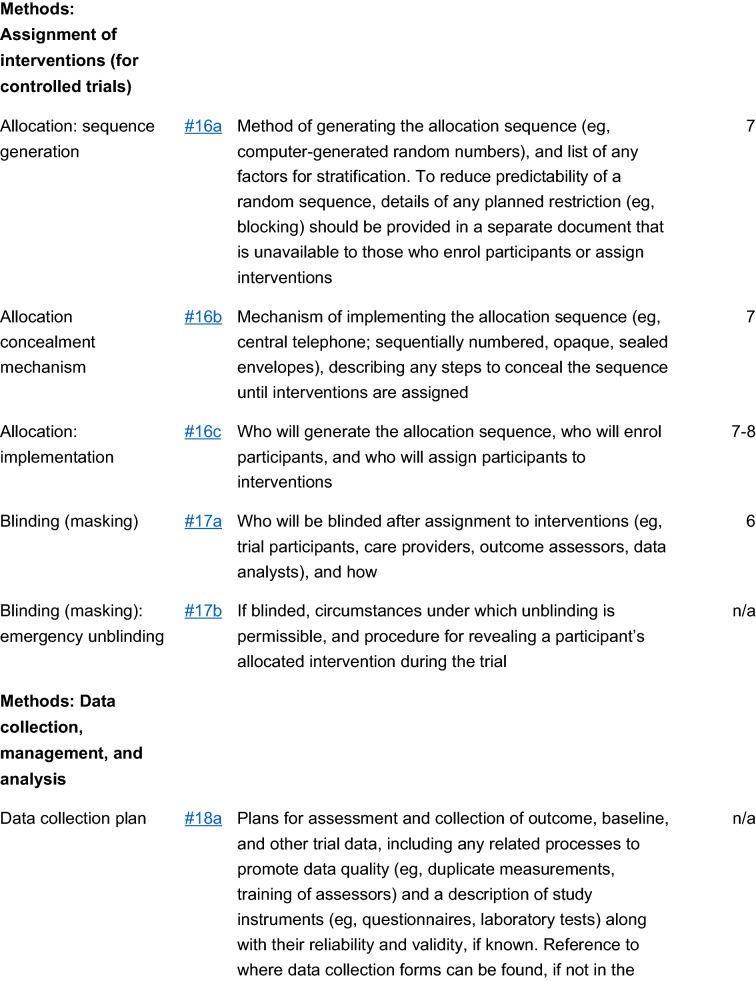

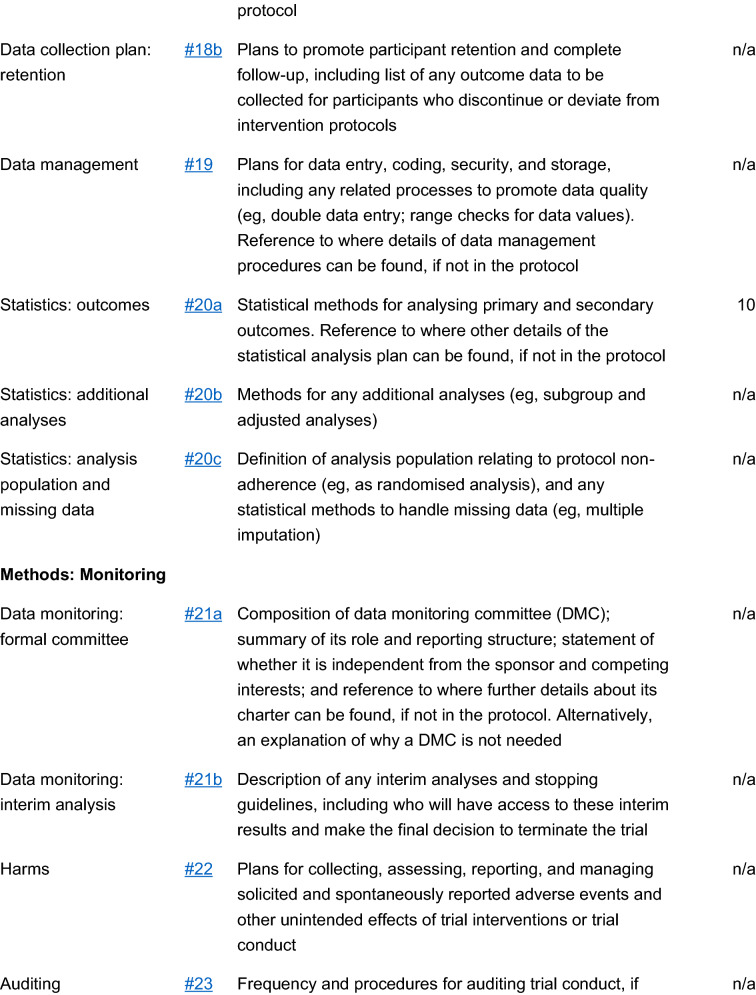

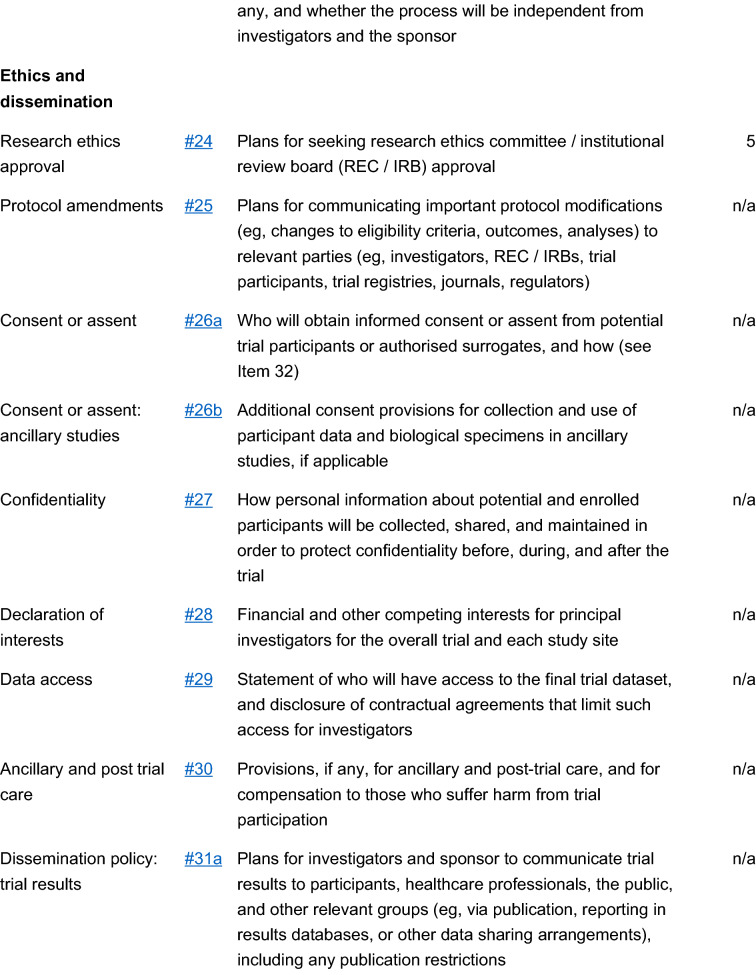

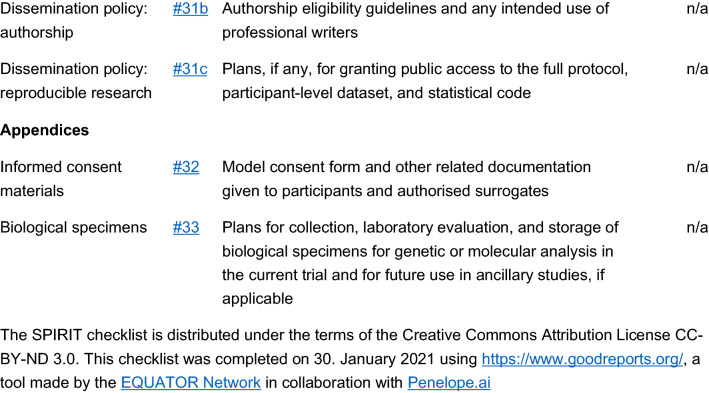
Spirit checklist for the study

The study is a multicenter, double blind, placebo-controlled, randomized, clinical trial designed to assess the efficacy and safety of varenicline (1 mg BID) in comparison to placebo for smoking cessation in diabetic smokers. The duration of active treatment will be 12 weeks and participants will be followed up in the nontreatment phase for an additional 40 weeks (52 week timepoint). The study will consist of an initial screening visit followed by a total of 18 study visits (12 ambulatory visits and 6 telephone contact visits) (Fig. 1). The present protocol followed the SPIRIT guidelines (Fig. 2). The study will be conducted in 6 diabetic outpatient clinics of five Italian hospitals in the province of Catania (Italy, Sicily).

### Study outcome measures

This study is intended to compare 12 weeks treatment with varenicline to matched placebo for safety and 12–52 weeks for smoking cessation efficacy in diabetic smokers.

#### Primary outcome measure

Consistent with previous varenicline randomized controlled trials (RCTs) [25–27] and the Society for Research on Nicotine and Tobacco recommendations [28], the primary efficacy end point is the carbon monoxide–confirmed (≤ 10 ppm) CAR from week 9 to week 24 (CAR 9–24 Wks). Smoking abstinence will be self-reported and validated by measurements of exhaled carbon monoxide levels with a calibrated handheld monitoring device (MicroCO, Vyare Medical, Inc.) during clinic visits.

#### Secondary outcome measures

Secondary efficacy end points included carbon monoxide–confirmed CARs for weeks 9 to 12 (CAR 9–12 Wks) and 9 to 52 (CAR 9–52 Wks) and 7-day point prevalence of abstinence at weeks 12, 24, and 52. Smoking abstinence will be self-reported and validated by measurements of exhaled carbon monoxide levels with a calibrated handheld monitoring device (MicroCO, Vyare Medical, Inc.) during clinic visits. Additional outcomes of interest will be: [1] Compare varenicline to placebo for changes in body weight and/or waist circumference at weeks 12, 24, and 52; [2] Compare varenicline to placebo for changes of diabetic outcomes (fasting blood glucose, HbA1c, insulinemia, and albumin to creatinine ratio) at weeks 12, 24, and 52; and (3) Compare varenicline to placebo for changes in blood pressure (BP) and heart rate (HR) at weeks 12, 24, and 52;

#### Safety end points

A summary table will present the number of events (includes lab data), number of subjects and severity of adverse events (AEs), and serious adverse events (SAEs). Any events documented in the period from the point of treatment initiation until 1 week after randomized treatment has been discontinued (V14) will be considered as relevant to the analysis.

#### Additional measures

Given that a better understanding of predictors of smoking cessation can be useful in identifying potential quitters and likely relapsers and that little is known about these predictors in diabetics, the role of different predictors of abstinence at week 12, week 24, and week 52 will also be examined. The level of smoking reduction will be also assessed in the participants who cannot achieved continuous abstinence at 12, 24 and 52 week.

### Randomization and interventions

#### Allocation to study treatment

Patients will be randomly assigned to either varenicline or placebo in a 1:1 ratio by using a computer-generated, 3-block randomization scheme.

#### Formulation and packaging

Varenicline (0.5 mg tablets) and placebo will be supplied in blinded bottles according to the computerized assignment. The hospital pharmacy will be in charge of randomization and packaging of the bottles.

#### Drug administration

Treatment will begin after the baseline visit.

Participants randomly assigned to varenicline will be titrated to full dose within the first week (0.5 mg/day for 3 days, 0.5 mg twice daily for 4 days; then 1 mg twice daily for the following 11 weeks). The day of up-dosing will correspond with the planned target quit date (TQD), one or two days before visit 2 at Week 1. Dosing should occur with a glass of water and it is recommended that subjects eat prior to dosing. There must be at least 8 h between the morning and evening dosing. If not tolerated, the dose may be reduced temporarily or permanently to 0.5 mg twice daily. The placebo tablets will look the same as the varenicline tablets. Dose titrations and allowable adjustments will be the same for both agents.

#### Compliance

Subjects will return blister cards at each programmed visit and a dosage record will be registered. Diabetic smokers will be subjected to their usual care throughout the study.

#### Cessation counseling

Both treatment groups will receive the same smoking cessation counseling starting at baseline and throughout the whole duration of the study. One-on-one counseling will be provided for up to 10 min and, whenever possible, done by the same counselor throughout the study.

### Study visits

The study will consist of a total of 19 visits: a screening visit, a baseline visit (V1), 12 study visits during the 12-week treatment phase (V2 to V13, of which 4 will be telephone contact visits) followed by an additional 5 study visits during the 40-week follow-up phase (V14 to V18, of which 2 will be telephone contact visits) (Fig. 1). Activities carried out during study visits are detailed in Table 1.

**Table 1:** Study schedule/flowchart

**Procedure**	Sc V0	BL V1	Wk 1 V2	Wk 2 V3	Wk 3 V4	Wk 4 V5	Wk 5 TC V6	Wk 6 V7	Wk 7 TC V8	Wk 8 V9	Wk 9 TC V10	Wk 10 V11	Wk 11 TC V12	Wk 12 V13	Wk 13 V14	Wk 24 V15	Wk 36 TC V16	Wk 44 TC V17	Wk 52 V18
Informed consent		X																	
Eligibility Criteria	X	X																	
Medical Hx	X	X																	
Antidiabetic drug use		X	X	X	X	X		X		X		X		X	X	X			X
Smoking Hx	X	X																	
Cigarette consumption	X	X	X	X	X	X	X	X	X	X	X	X	X	X	X	X	X	X	X
Exhaled CO		X	X	X	X	X		X		X		X		X	X	X			X
BP, HR	X	X	X	X	X	X		X		X		X		X	X	X			X
BMI, waist		X												X		X			X
Sociodemographics		X																	
Motivation to quit by VAS		X																	
FTCD		X																	
BDI & BAI		X																	
Self-Efficacy to quit by VAS		X																	
Cessation counseling		X	X	X	X	X	X	X	X	X	X	X	X	X	X	X	X	X	X
Adverse events		X	X	X	X	X		X		X		X		X					
Blood chemistry and CBC	X	X		X											X				
Urinalysis	X	X		X											X				
DM lab tests		X													X	X			X
Dispense study drug*		X	X	X	X	X		X		X		X							
Dosing record			X	X	X	X		X		X		X		X					

*Sc* screening, *BL* baseline, *TC* telephone contact, *CO* carbon monoxide, *HR* heart rate, *BP* blood pressure, *BMI* body mass index, *VAS* visual analog score, *FTCD* Fagerstrom Test for Cigarette Dependence, *BDI* beck depression inventory, *BAI* beck anxiety inventory, *QoL* quality of life, *CBC* complete blood count, *DM* diabetes mellitus

*Dispense study drug – a week supply of drug/placebo is dispensed at each visit with the exception of Wk 4, Wk 6, Wk 8 and Wk 10 when a whole 2 weeks supply is given

#### Screening visit

At screening, patients who expressed an interest to participate in the study, will be checked for eligibility by evaluating the criteria for inclusion and exclusion. Medical history, and smoking history will be noted. Physical examination will be performed. BP and HR will be measured. Blood and urine will be collected for standard clinical laboratory evaluations (including HbA1C). Qualified participants will be then invited to attend the Baseline visit.

#### Baseline visit (V1)

The Baseline Visit will be scheduled no less than 3 working days after screening so that laboratory results would be available for review. Patients with abnormal laboratory results judged to be clinically significant will not be considered for inclusion. Eligible patients are asked to go over the patient information sheet and to sign the consent form. General socio-demographic features, medical hx, drug prescriptions, smoking hx, and motivation to quit will be noted in the chronic renal failure (CRF). The following baseline assessments will be carried out: number of cigarette smoked/day, eCO levels, blood pressure, HR, BMI, waist circumference, and questionnaires (Fagerström Test for Cigarette Dependence (FTCD), Beck Depression Inventory (BDI), Beck Anxiety Inventory (BAI)). Blood and urine samples will be collected for standard clinical laboratory evaluations (including fasting blood glucose, HbA1C, insulinemia, and albumin to creatinine ratio). Any AEs will be noted in the CRF. Eligibility criteria will be reviewed again prior to randomization. Regardless of their assignment, all patients will receive smoking cessation counseling and instructed to set a TQD 1–3 days before the next study visit (visit 2 at Week 1). Patients will receive a full week supply of their assigned treatment prior to check-out (either varenicline or placebo depending on the study-arm allocation). Study drug will be dispensed according to the plan illustrated in Table1.

#### Treatment study visits 2 to 13

After V1, patients will return for their treatment phase visits (V2–V13). With the exclusion of Visits 6, 8, 10 and 12 (telephone contact visits), patients will return to attend weekly visits to the clinic over the following 12 weeks. Study visits will occur within 3 days of each scheduled visit date. Patients returning for their Week-1 visit (i.e. V2; TQD-visit) must have adhered to their TQD, abstaining from smoking for 1–3 days before the study visit. At each visit, patients will be reminded of the importance of staying cigarette free and will receive professional counseling for relapse prevention. Number of cigarette smoked/day, eCO levels, blood pressure, HR (BMI, and waist circumference only at Week-12 visit), and AEs will be noted in the CRF. Any unused study drug will be returned to the principal investigator. Patients will receive a supply of their assigned treatment prior to check-out according to the plan illustrated in Table1.

#### Follow-up study visits 14 to 18

Following completion of the treatment phase (V2–V13), patients will continue in the nontreatment follow-up phase of study. Only patients completing the week-12 visit will be eligible to continue in the nontreatment follow-up phase (V14–V18). With the exclusion of Visits 16 and 17 (telephone contact visits), patients will return for their study visits at week-13 (V14), week-24 (V15), and week-52 (V18). Number of cigarette smoked/day, eCO levels, BP and HR (BMI, and waist circumference only at week-24 and week-52 visits) will be noted in the CRF. Blood and urine samples will be collected for evaluation of fasting blood glucose, HbA1C, insulinemia, and albumin to creatinine ratio at week-24 and week-52 visits.

### Study assessments

Besides lab tests (for blood chemistry—including sodium, potassium, chloride, BUN, creatinine, glucose, SGOT, SGPT, LDH, alkaline phosphatase, total bilirubin, total cholesterol, HDL, LDL triglycerides, complete blood count—urinalysis, and T2DM specific lab tests—including fasting glucose, HbA1c, insulinemia, and albumin to creatinine ratio), the following assessments will be performed throughout the study:

#### Exhaled carbon monoxide monitoring

Participants’ smoking status will be objectively verified by measurements of exhaled carbon monoxide levels with a calibrated handheld monitoring device (MicroCO, Vyare Medical, Inc.). Levels of CO in the exhaled breath of a regular smoker (eCO) are generally ≥ 10 ppm. Participants will not be allowed to smoke within 30 min prior to eCO level measurements.

#### Office BP and HR measurements

Systolic (SBP) and diastolic BP (DBP) measurements will be conducted according to the recommendations of the Seventh Report of the Joint National Committee on Prevention, Detection, Evaluation, and Treatment of High Blood Pressure [29]. After a 5-min rest, BP and HR measurements will be obtained by a semi-automated oscillometric sphygmomanometer (Smart Pressure, CA-MI Snc, Parma, Italy). Two measurements in the sitting position, spaced 1–2 min apart, will be obtained. Measurements will be taken late in the morning, and participants asked not to smoke or consume caffeinated drinks for at least 30 min prior to each visit. The average of two measurements will be considered for analysis.

#### BMI and waist circumference

Participants will remove shoes and heavy clothing and will be weighed using a mechanical column scale (Seca, Intermed Srl, San Giuliano Milanese, Italia). Height measurements will be taken by using a standing scale slide bar. BMI will be computed as weight/height^2^ (kg/m^2^). To correctly measure waist circumference, place a tape measure just above the hipbones, then bring the tape measure all the way around the body until leveling to the belly button. Make sure tape measure is not too tight and that it's straight, even at the back. Check the number on the tape measure right after breathing out.

#### Fagerström test for cigarette dependence (FTCD)

Cigarette dependence will be assessed via a FCTD questionnaire [30]. The questionnaire consists of 6 questions which will be answered by the participant himself/herself. The scores obtained on the test allow to classify nicotine dependence into 3 levels: Mild (0–3 points), moderate (4–6 points), and severe (7–10 points).

#### Beck depression inventory

Subjective ratings of depression will be assessed with Beck Depression Inventory–II (BDI-II) [31]. The BDI-II is a 21-item, self-report rating inventory that measures characteristic attitudes and symptoms of depression. Internal consistency for the BDI-II ranges from 0.73 to 0.92 with a mean of 0.86. The BDI-II demonstrates high internal consistency, with alpha coefficients of 0.86 and 0.81 for psychiatric and non-psychiatric populations, respectively. Scores from 0 through 9 indicate no or minimal depression; scores from 10 through 18 indicate mild to moderate depression; scores from 19 through 29 indicate moderate to severe depression.

#### Beck anxiety inventory

Subjective ratings of anxiety will be assessed with Beck Anxiety Inventory (BAI) [32]. The BAI evaluates physiological and cognitive symptoms of anxiety. Each of the 21 BAI items is descriptive of a symptom of anxiety and is rated on a scale of 0 to 3. The BAI can be administered verbally by a trained interviewer or can be self-administered. The BAI has been found to discriminate well between anxious and non-anxious diagnostic groups and, as a result, is useful as a screening measure for anxiety in a variety of clinical populations. It has an average reliability coefficient of 0.92 and a test–retest reliability of 0.75. Scores from 0 through 7 is interpreted as a "Minimal" level of anxiety; 8–15 as "Mild"; 16–25 as "Moderate", and; 26–63 as "Severe".

### Statistical methods

#### Sample size determination

The sample size calculation for this RCT was based on success quit rates from previous smoking cessation studies [21, 33, 34]. We computed that 174 subjects (87 per each study arm) will be required to have 90% power with two-sided 0.05 significance level test to detect a difference of at least 18.7% between treatment groups for CAR 9–24 weeks. Allowing for a conservative attrition rate of approximately 40%, the target number of participants will be increased to a total of 300 (150 per each study arm).

#### Methods of analyses

Baseline and demographic data will be listed for all treatment groups. Summary statistics will be provided for each treatment group. At baseline, differences between varenicline and placebo groups will be evaluated by means of one-way analysis of variance and Mann–Whitney *U*-test for normally and not normally distributed continuous variables, respectively; *χ*^2^ test will be used for categorical variables. The secondary endpoints will be analyzed using procedures similar to that described above for the primary endpoint. Intention-to-treat analysis will be adopted for efficacy evaluation, assuming that subjects lost to follow-up continued smoking.

Safety data will be summarized for both treatment groups and summary statistics provided.

Any events documented in the period from the point of treatment initiation until 1 week after randomized treatment has been discontinued (V14) will be considered as relevant to the safety analysis. AEs will be coded using MedDRA (Medical Dictionary for Regulatory Activities) and assigned grades based on NCI CTCAE (National Cancer Institute Common Terminology Criteria for Adverse Events). Between-groups comparisons for both individual and clustered AE rates will be carried out using χ^2^ testing or Fisher’s exact test, as appropriate.

Multiple logistic regression models will be built to identify independent predictors associated with CARs at week 12, week 24, and week 52.

### Safety reporting

#### Adverse events

All observed or volunteered AEs, regardless of treatment group or suspected causal relationship to study drug, will be recorded. This includes symptoms thought to be related to withdrawal from nicotine. Events involving adverse drug reactions, illnesses with onset during the study, or exacerbations of pre-existing illnesses should be recorded. Exacerbation of the disease under study (type 2 diabetes), is defined as a manifestation (sign or symptom) of the illness that indicates a significant increase in the severity of the illness as compared to the severity noted at the start of the trial. It may include worsening or increase in severity of signs or symptoms of the illness, increase in frequency of signs and symptoms of an intermittent illness, or the appearance of a new manifestation/complication. Exacerbation of a pre-existing illness should be considered when a subject requires new or additional concomitant therapy for the treatment of that illness during the trial. In addition, clinically significant changes in physical examination findings and abnormal objective test findings (e.g., laboratory) should also be recorded as AEs. For all AEs, the investigator must pursue and obtain information adequate both to determine the outcome of the AE and to assess whether it meets the criteria for classification as a SAE. For all AEs, sufficient information should be obtained by the investigator to determine the causality of the AEs (i.e., study drug or other illness).

#### Serious adverse events

All SAEs (as defined below) regardless of treatment group or suspected relationship to study drug must be reported immediately. A SAE is any adverse drug experience occurring at any dose that:

1. Results in death;

2. Is life-threatening;

3. Results in inpatient hospitalization or prolongation of existing hospitalization;

4. Results in a persistent or significant disability/incapacity.

Any SAE or death must be reported immediately independent of the circumstances or suspected cause if it occurs or comes to the attention of the investigator at any time during the study through 30 days after the last administration of study drug. Any SAE occurring beyond 30 days after the last administration of study drug must be promptly reported if a causal relationship to study drug is suspected.

#### Clinical laboratory parameters and abnormal laboratory test results

The results of all laboratory tests required by the protocol will be recorded. All clinically important abnormal laboratory tests occurring during the study will be repeated at appropriate intervals until they return either to baseline or to a level deemed acceptable by the investigator or until a diagnosis that explains them is made. The criteria for determining whether an abnormal laboratory test result should be reported as an AE are as follows:

1. Test result is associated with accompanying symptoms, and/or.

2. Test result requires additional diagnostic testing or medical/surgical intervention, and/or.

3. Test result leads to a change in study dosing or discontinuation from the study, significant.

additional concomitant drug treatment or other therapy, and/or.

4. Test result leads to any of the outcomes included in the definition of a SAE, and/or 5. Test result is considered to be an AE by the investigator.

The following tests will be completed at the screening and baseline visits: blood chemistry (including sodium, potassium, chloride, BUN, creatinine, glucose, SGOT, SGPT, LDH, alkaline phosphatase, total bilirubin, cholesterol, and triglycerides) and complete blood count. In the event of clinically significant abnormalities, urine samples will be sent for urinalysis. Moreover, the following additional lab tests will be carried out at screening, baseline, week 13, week 24 and week 52 (HbA1c, fasting glucose, total cholesterol, HDL, LDL, triglycerides, insulinemia, albumin to creatinine ratio, and plasma creatinine).

## Results

In total, 300 subjects have been randomized to the varenicline (*n* = 150) or placebo (*n* = 150) groups with 194 subjects completing the study at week 52; 100 in the varenicline group and 94 in the placebo group. The conduct phase of the study is completed, while data cleaning and analyses are ongoing.

## Discussion

Smoking and diabetes is a dangerous liaison and promoting smoking cessation for those with diabetes must be a top priority [8]. There is a pressing need for efficient interventions to reduce or prevent morbidity and mortality in smokers with DM. The high-affinity α4β2 nicotinic acetylcholine receptor partial agonist varenicline has been shown to to be effective for smoking cessation in smokers [19, 20], including those with cardiovascular disease [21].

COPD [22], or depression [23]However, there is very limited data about its efficacy and safety in smokers with diabetes [35], and a clinical trial of varenicline targeted specifically at smokers with T2DM is warranted to better inform medical/health authorities (FDA, EMEA) and physicians worldwide. To gather such evidence we will conduct the first randomized, double blind, placebo-controlled study of efficacy and safety of varenicline (1 mg BID) in smokers with T2DM.

The study protocol adopts a number of measures that contribute to quality of the study.

The gold standard to rigorously assess smoking cessation success in drug trials is continuous abstinence. Consistent with previous varenicline RCTs [25–27] and the Society for Research on Nicotine and Tobacco recommendations [28], we have adopted.

CAR as a robust primary efficacy end point. Although smoking abstinence will be self-reported, objective biochemical validation by measurements of exhaled carbon monoxide levels with a calibrated CO meter will be mandatory at each study visit.

The RCT study design with extended follow-up at 52 weeks will provide a robust answer to determine the safety and long-term efficacy of varenicline in T2DM. The length of the study was based on the consideration that substantial changes in the primary endpoint (CAR 12-week) could be reasonably observed early in the course of the trial. It is however likely that a much longer follow-up period is necessary to firmly establish findings consistency over time, hence study duration is extended to 52 weeks. Clearly, randomization will equalize variation in smoking history and other variables between study arms, thus ensuring high quality data.

Importantly, the entire study is designed keeping the welfare of all participants at its center; at every contact participants will be counseled to stop smoking by experienced clinical psychologists. Therefore, the potential long-term benefit of participating in this study is—under a best case scenario—to completely stop smoking even after completion of study follow-ups.

Compliance with the study protocol is critical as failure to regularly take varenicline would reduce or nullify the expected quit rates as well as produce inaccurate safety reporting. Besides, being instructed on the importance of adhering to their randomized product allocation, participants will be asked to report any non-compliance via a study diary and will be informed that biochemical verification of compliance and assessments checks of adherence will be conducted at each clinic visits. Strict adherence to the study protocol should be also aided by frequent weekly visits for drug checks, during which participants will be asked to return all empty, part-used, and unused blinded bottles (containing study drugs/placebo). Any non-compliance will be recorded in the study diary after counting all empty, part-used and unused bottles. Although not expected that compliance for this study will be materially different compared to other comparable studies, our power calculations are over-estimated to take this into account. Of note, non-compliance to study products is in itself an interesting outcome and we will be able to assess the impact of different level of non-compliance on smoking cessation rates.

Our study has limitations and may face challenges.

First, due to the relatively long duration of the study (52-weeks), maintaining a sufficient level of subject retention may be a challenge. Nonetheless, trial attendance and retention is likely improved by inviting participants to return to the clinic for their free supply of study drugs, by offering a dedicated fast track approach for their outpatient clinic appointments and by providing tailored counseling.

Second, the results cannot be generalized to all diabetic patients who smoke. We will recruit a (ambulatory) population of diabetic smokers who have been stably treated for T2DM. Therefore, the study protocol excludes smokers with untreated disease and T1DM smokers. Moreover, the study protocol excludes smokers with diagnosed depression or taking antidepressant medications. Therefore, the study cannot address the safety or efficacy of varenicline in smokers with comorbid depression, which occurs at a higher rate in smokers with diabetes than in the general population of smokers [36].

The results of this RCT with varenicline will help determine whether an optimally varenicline-treated cohort of T2DM patients who smoke will experience significant success rates, without causing side effects.
